# Bioactive Compound Fingerprint Analysis of Aged Raw Pu’er Tea and Young Ripened Pu’er Tea

**DOI:** 10.3390/molecules23081931

**Published:** 2018-08-02

**Authors:** Vasilisa Pedan, Sascha Rohn, Mirjam Holinger, Tilo Hühn, Irene Chetschik

**Affiliations:** 1 Zurich University of Applied Sciences, Life Sciences and Facility Management, 8820 Wädenswil, Switzerland; tilo.huehn@zhaw.ch (T.H.); irene.chetschik@zhaw.ch (I.C.); 2 Hamburg School of Food Science, Institute of Food Chemistry, University of Hamburg, Grindelallee 117, 20146 Hamburg, Germany; rohn@chemie.uni-hamburg.de; 3 Research Institute of Organic Agriculture (FiBL), Ackerstrasse 113, 5070 Frick, Switzerland; mirjam.holinger@fibl.org

**Keywords:** Pu’er tea, LC-MS analysis, polyphenols, principal component analysis, hierarchical cluster analysis, brewing condition

## Abstract

Pu’er tea produced from *Camellia sinensis* var. *assamica* is a widely appreciated and consumed beverage that can be divided into two kinds of tea depending on the different fermentation processed used, the special sensory characteristics, and their chemical composition. However, authentication seems to be very important for such teas, as they are traded to comparatively high prices, especially in Europe. The results for selected biochemical markers showed that aged raw pu’er tea contained 210.2 mg GAE/g polyphenols, of which 2.2 mg/g were gallic acid, 16.1 mg/g theogallin, 35.1 mg/g (−)-epigallocatechin gallate, and 40.1 mg/g (−)-epicatechin gallate, on average. Young ripened pu’er tea contained about 104.6 mg GAE/g polyphenols, of which 5.5 mg/g gallic acid, 0.9 mg/g theogallin, 0.7 mg/g (−)-epigallocatechin gallate, and 1.8 mg/g (−)-epicatechin gallate, on average. An additional objective of the present study was to unravel the best brewing conditions for optimal extraction of the bioactive compounds. Infusions of nineteen commercial teas (from pu’er cakes) were obtained at different time-temperature ratios for studying the content of bioactive compounds (flavan-3-ols, flavonols, caffeoylquinic acids, methylxanthines). Brewing at 90 °C for 5 min was the best condition to obtain a high content of total polyphenols in ripened pu’er tea. Principal component analysis and hierarchical cluster analysis showed, that young ripened and aged raw pu’er tea can be successfully differentiated by the analyzed chemical compounds. Principal component analysis results indicated that young ripened pu’er tea has higher contents of gallic acid, quercetin, and kaempferol than aged raw pu’er tea.

## 1. Introduction

Many myths are told concerning the history of pu’er tea. One of them tells of the discovery by the Chinese emperor Shennong (2737 BC), who drank boiled water under a tree and a dead leaf from the wild tea bush fell into the water. The infusion turned into a brownish color, whereby the outcome was appreciated as a refreshing and aromatic beverage. During the Tang dynasty (618–907 AC), tea became a commercial product, and tea trade began between the producing region in Yunnan, China and the surrounding areas, the method of pu’er tea preparation was written down, and pu’er tea was used as a drug with medicinal effects [1].

Due to its sensory attributes and sociocultural factors, pu’er has gained high popularity, not only in China, but also in Europe. Nowadays, it is appreciated as luxury and health-promoting ‘superfood’, especially because of its antioxidant activity [2] and its lipid-lowering effect [3]. A study done by Lu and Hwang [4] investigated thirty samples of pu’er tea and analyzed for cholesterol synthesis inhibitory activity in a HepG2 cell model with an inhibition ratio ranging from 7% to 35%. According to a work done by Kuo and co-workers [5], 4% of pu’er tea leaves increased the lipoprotein level of HDL-C and decreased the level of LDL-C by oral intake, whereby other teas such as black tea (BT), green tea (GT), and oolong tea (OT) only decreased the levels of both. One widely believed assumption amongst pu’er tea lovers, is the weight loss property based on the suppressing effect of the fatty acid synthase, as shown in rat liver. The results further showed that the suppression of body weights of tea leaves-fed rats were in the order: OT > pu’er tea > BT > GT. Pu’er tea and OT could lower the levels of triglyceride more significantly than that of GT and BT [5].

Depending on its morphological proximity, pu’er tea is more associated to a subspecies of the *Camellia sinensis* var. *assamica* called *qingmao*, which is a larger, light green, non-serrated leaf, quick growing tea tree variety, grown in a temperate, frost-free, and regular rainfall throughout the year. Qingmao pu’er tea is presented in two varieties: raw pu’er tea (sheng) and ripened pu’er tea (shu) according to the processing technology and the quality characteristics. Both varieties are suitable to ageing and are therefore available on the market as both young and aged tea. The leaf processing resulting in ripened pu’er was developed in the seventies in an effort of reproducing in short time (few months) the taste of aged raw pu’er (several years old). This study compares the bioactive compounds of aged raw pu’er (APT) and young ripened pu’er (YPT) highlighting differences in the chemical composition of the two varieties, which have similar sensory characteristics. Contrary to other teas, non-fermented (green tea), semi-fermented (oolong tea), and fermented (black tea), pu’er tea is known as post-fermented tea. Pu’er tea is made from sun-dried tea leaves, often compressed into a final cake. Herein, APT undergoes a natural aging process with further microbial post-fermentation during storage at room temperature and normal humidity [6]. Compared to the other teas, APT has a long storage time (up to decades) before consumption. YPT is made from sun-dried tea leaves as most GT from the same region (Yunnan, China). However, with an accelerated ageing process known as wet piling, tea leaves are spread on trays, where water is sprayed on them. The trays are kept at higher temperatures (40–60 °C), humidity, and a fermentation time of 1–2 months [7,8]. Under these conditions, the speed and degree of oxidation are higher than for APT [9], whereby all flavan-3-ols in YPT are significantly reduced compared to those of APT.

Although there are plentiful studies on the chemical composition of BT and GT, the chemical constituents of pu’er tea and their transformation have not been characterized comprehensively, so far. Studies done by Zhang et al. [10] covered most of the commercial teas on the market and provided a clear description of the chemical constituents of APT and YPT. Nevertheless, to understand the differences of the major compounds and to exclude coincidences between APT and YPT, investigations must be extended to a higher representative sample number. Moreover, authentication is very important for products that are sold with advertisements promising certain health-beneficial effects, as food fraud cannot be excluded for luxury products and ‘exotic’ superfoods, especially in Europe. So, the analytical differentiation of APT and YPT was the initial aim of this study. Characterization of the plant material used in the study is presented in Table 1.

Besides characterizing the biochemical composition of the tea leaves for differentiation, it is necessary to implement investigations on the preparation of the final beverages. Therefore, the present study investigated the effect of brewing water on the chemical composition of tea infusions containing different concentrations of bioactive compounds. The aqueous extraction of flavan-3-ols depends on time and temperature, so monitoring these parameters, while making an infusion is of great importance to understand the kinetics of valuable compounds and therefore, potential health-benefits [11]. Different researchers have studied the extraction kinetics of catechins from white tea (WT), GT, and BT based on water temperature and extraction time [12], but not yet for pu’er tea. Therefore, a further aim of this research was to study the effect of the extraction time and temperature on the release of the valuable molecules into the pu’er infusion, in relation to their initial content in the leaves. As such, the objectives of this study were on the one hand to investigate the differentiation/authentication between APT and YPT with regard to the content and composition of catechins, flavonols, and their derivatives, as well as studying the effects of extraction according time and temperature and thus, to select the most suitable time-temperature conditions for tea brewing.

## 2. Results and Discussion

### 2.1. Identification and Quantification of Chemical Components Using LC-DAD/ESI-MS

In this study, ‘tea leaves’ extracts’ were used for the identification of the biochemical composition of apt and YPT. Pu’er polyphenols were characterized and quantified using external standards. Twenty-one compounds were identified based on LC-DAD/ESI-MS analysis with regard to retention time, absorbance spectra, and mass fragmentation pattern (Table 2). In general, both tea variants showed a characteristic chromatographic phenolic profile. Catechins and their derivatives were the most abundant compounds detected. Besides flavonols, also methylxanthines such as caffeine and theobromine were found in the extracts. Other substances were only at trace levels. The chemical composition of both tea variants was similar. However, the levels of most of the compounds were higher in APT (Table 2).

#### 2.1.1. Flavan-3-ols

The difference in flavan-3-ols content between the both tea variants was significant. The most abundant polyphenols in APT were catechins and their derivatives in the following order: ECG > EGCG > EC > EGC > GCG > C > GC > CG. However, differences in flavan-3-ol content between origins were comparatively low. As shown in Table 3, all of the analyzed flavan-3-ols were lower in YPT. The present study shows the high amount of especially ECG and EGCG in APT compared to YPT. The critical review done by Harbowy and co-workers [13] describes that during the fermentation of pu’er tea, flavan-3-ols are oxidized and condensed to larger phenolic compounds such as theaflavins and thearubigins. This effect also results in a darkening color of the tea leaves and decreasing astringency with increasing fermentation time.

The high content of GC in YPT can be explained by the biotransformation of EGCG to EGC and further on to GC [14]. Studies done by Zhang et al. [10] already gave a broad understanding about chemical composition of APT and YPT. Therein, they stated that APT contains a significant higher (*p* < 0.01) amount of EGCG, C, EC, EGC, GCG, and ECG than YPT, with the following order: ECG (9.86 mg/g) > EGCG (9.43 mg/g) > EC (6.01 mg/g) > EGC (5.00 mg/g) > GC (3.64 mg/g) > C (3.16 mg/g) > GCG (1.37 mg/g) for APT.

Work done by Yi et al. [9] stated that ECG is a unique component in the chemical composition of APT, which also was confirmed in the present study. According to the cited study, none of the other tea variants had a comparable high content of ECG than APT with 30.60 ± 4.18 mg/g, e.g., GT (17.10 ± 3.34), YT (16.23 ± 7.01), WT (8.12 ± 3.05), OT (5.09 ± 1.64), BT (2.65 ± 2.25) and YPT (1.57 ± 2.46). The present study could also confirm a significant difference of ECG in APT with 40.14 ± 5.76 compared to YPT with 1.76 ± 0.33 mg/g. However, ECG was the major catechin in all teas, accounting for about one third of the total catechins. Lv et al. [7] stated in their study that ECG content in APT can be compared with the content of black tea. Furthermore, the present study showed a significant decrease of flavan-3-ols in YPT, being accompanied with an increase of GA. In 1998, Lin et al. [15] focused on the content of catechins, GA, and methylxanthines in fully fermented pu’er teas. In that study, they determined a very low level of catechins and a high level on GA. That study observed an increase of GA during the fermentation owing to its liberation from catechin gallates. It can be generated during the physical bruising stage of the processing procedure [9]. Therefore, GA is the most important phenolic acid in pu’er tea. Analyses done by Zhang et al. [10] detected a GA content of 1.47 mg/g for APT and 6.51 mg/g for YPT.

Duh et al. [16] stated that the content of total catechins in tea is related to the degree of fermentation of tea, which implies that the higher the degree of the fermentation, the more intense is the decrease in total catechins.

Pu’er teas are consumed because of their health-beneficial effects hypothesized. According to Lu and Hwang [4], the tea polyphenols show an inhibitory effect on cholesterol biosynthesis with their activity decreasing in the following order: GCG > EGCG > ECG > GA > EGC > myricetin > Q > C > EC. Kaempferol showed no dose-dependent activity in inhibiting the biosynthesis of cholesterol in the HepG2 cell model used. Furthermore, authors of that study stated that catechins with a galloyl structure in the B ring or a GA moiety in the structure will have a better inhibitory activity.

However, older tea leaves of APT and YPT have been characterized much less. This is not only because of their rare occurrence, but also because of their high price. In contrast, for the present study samples were available with sheng produced in 1980 and 1992, and shu produced in 1980 and 1994, respectively. However, differences between HPLC chromatograms of the various samples were not apparent by visual examination. In Table 3 and Table 4 it is shown that older pu’er tea samples differ in their concentration of biochemical compounds. The common opinion that longer aging periods improve the quality of pu’er tea cannot be supported by the present study. Nevertheless, a smoothing character goes along with longer storage time because of a continuous decrease of typical bitter and astringent chemical constituents [10].

#### 2.1.2. Flavonols

Flavonol aglycons such as quercetin and kaempferol and glycosides of both, namely Q-gal, Q-glu, K-rut, and K-glu have been tentatively identified in APT and YPT. The distribution of these phenolic compounds was similar in all samples, whereby also in this case the content on flavonol glycosides was consistently higher in APT than in YPT (Table 4). The following order of concentration of flavonol glycosides was observed for APT: K-rut > Q-glu > Q-gal > K-glu. In addition, flavonols glycosides in YPT were identified in the following descending order: K-rut > Q-glu > K-glu > Q-gal. However, the aglycons were more present in YPT than in APT, with a content of quercetin (98 µg/g) and kaempferol (46 µg/g) in APT, respectively quercetin (276 µg/g) and kaempferol (98 µg/g) in YPT. 

Natural fermentation processes are based on a high natural diversity of microorganisms, often uncharacterized with regard to metabolism of secondary plant metabolites. Hur et al. [17] reported an increase of phenolic compounds and flavonoids during fermentation in plant-based food, which is the result of a microbial hydrolysis reaction. During fermentation, the enzyme β-d-glycosidases can catalyze the hydrolysis of inter-sugar linkages, releasing the glycosides and the aglycons [18]. This enzyme is considered as a possible reason for the deglycosylation of kaempferol-3-*O*-glucoside into kaempferol by fermentation with *Bifidobacterium pseudocatenulatum* B7003 [19] or *Aspergillus awamori* splitting Q-glu into Q [20]. When trying to find microbial pathways, Huynh et al. [21] postulated that microorganisms can be responsible for further diverse conversion reactions of phenolic compounds, so is *Lactobacillus plantarum* converting Q-glu to Q [22].

Aglycons are hypothesized to be more antioxidant than their corresponding glycosides. Also the absorption in small intestine and colon depends to a certain extent on the chemical structure. Aglycons can be, due to their comparatively higher hydrophobicity, more easily absorbed by the small intestine, while flavonoid glycosides have to be converted into aglycon form [23], therefore enhancing the digestibility and bioactivity.

With regard to health beneficial properties, a study done by Zhao et al. [24] showed for fermented pu’er tea a more potent anticancer activity (85% inhibition) than for an unfermented pu’er tea (67% inhibition). In that study, the effect was traced back to higher concentrations of gallic acid, resorcilic acid, but especially quercetin and kaempferol levels in the fermented pu’er teas. However, it should be noted that the terminology fermented pu’er tea is often used as a synonym for ripened pu’er tea.

However, traces of theophylline, theaflavin, luteolin, luteolin-6-*C*-glucoside, quercetin-3-*O*-rutinoside, quercetin-3-*O*-glucuronid, apigenin, kaempferol-3-*O*-arabinoside, and theaflavin were not detectable in the UV-chromatogram, but in the total ion signals of the mass spectrometer detector (Table 2). Some of them are described in the literature [25,26].

#### 2.1.3. Caffeoylquinic Acids

Caffeoylquinic acids were identified in both tea variants containing mainly esters of caffeic acid and (−)-quinic acid such as 3-CQA, 4-CQA, and 5-CQA. The present study showed the following order of caffeoylquinic acids in APT extracts: 4-CQA > 3-CQA > 5-CQA, whereas 3-CQA was more prominent in YPT (Table 4).

A profiling study done by Zhao et al. [25] investigated and tentatively identified components in young raw pu’er teas using UPLC-DAD-MS. Among the five green pu’er teas, an average content of 3-CQA with 138 µg/g, 4-CQA with 5190 µg/g, and 5-CQA with 407 µg/g was detected. The contents of caffeoylquinic acids were much lower in GT with 3-CQA with 43 µg/g, 4-CQA with 813 µg/g and 5-CQA with 90 µg/g, respectively in WT with 3-CQA with 44 µg/g, 4-CQA with 813 µg/g and 5-CQA with 92 µg/g.

The antioxidant potential of caffeoylquinic acids has been reported in the extracts of various plants. Especially 5-CQA, as a widely available dietary phenolic compounds, has been reported to exhibit health advantages. Studies indicated that consuming coffee could significantly reduce blood pressure in patients with mild hypertension [27]. Treatment of caco-2 cells with 5-CQA and 3-CQA from prune showed a significant effects on cell proliferation and cell morphology [28]. These findings of cell proliferation inhibition suggest that both 5-CQA and 3-CQA could be cancer preventive components.

#### 2.1.4. Methylxanthines

Fermentation process did not affect the contents of methylxanthines. Caffeine (30.8 mg/g) and theobromine (1.2 mg/g) in APT were almost equivalent compared to those in YPT (30.2 mg/g for caffeine and 1.6 mg/g for theobromine). These findings are in accordance with descriptions from Zhao et al. [25] who analyzed a caffeine content of 24.1 mg/g and a theobromine content of 2.1 mg/g. However, a study done by Zhang et al. [10] showed a significant difference (*p* < 0.01) in the content of caffeine between APT with 11.4 mg/g and YPT with 15.5 mg/g, whereas the content of theobromine remained unaffected with 0.8 mg/g in APT, respectively 0.9 mg/g for YPT. Unno et al. [29] suggested that the components of pu’er tea leaves are oxidized by enzymatic reaction during fermentation, whereas in return substances can be formed during fermentation process. The constituents of pu’er tea have been the subject of intensive investigations of Lin et al. [15]. Analyzing seven YUNNAN teas, an average of caffeine with 7.70 ± 0.23 mg/100 mg dry tea leaves and theobromine content with 0.63 ± 0.09 mg/100 mg has been determined using HPLC.

Wang et al. [8] reported for infusions of sun-dried pu’er tea a significant higher amount (*p* < 0.5) for caffeine and theobromine than for ripened pu’er tea. Pu’er tea made by submerged fermentation possessed a high caffeine (135.2 mg/g) and theobromine content (6.4 mg/g), whereby pu’er tea made by solid-state fermentation process contained a lower caffeine (96.5 mg/g) and theobromine content (4.5 mg/g). This was explained by the inability of *Aspergillus tubingensis* used in submerged fermentation to metabolize caffeine and theobromine, whereby solid-state fermentation depends on a mix of microorganisms, which can metabolize caffeine and theobromine to other compounds, also via the so called cross-feeding, where some species can use the degradation products of other microorganisms present.

Further alkaloids such as theacrine, as one of the major purine alkaloids in the leaves of *Camellia assamica* var. *puanensis* (puan tea) could not be detected in the present study.

In summary, the high catechin contents in APT leaves can be explained by the processing method. In detail, starting from plucking, fresh tea leaves are subjected to a withering process, which removes excess water from the leaves and ensures an inactivation of polyphenol oxidases. By the following rolling process, leaves are getting rolled and twisted rupturing the cell and release of the cell fluid, promoting fermentation. The characteristic of pu’er tea is sun drying, for getting rid of the remaining moisture. Afterwards, loose leaves are often steamed for a few seconds to soften the leaves and to allow the leaves to stay together when pressed. After compressing to various shapes, APT is left ageing at ambient temperatures, in some cases for decades. In this period, also oxidation can occur resulting from the enzymatic activity of microorganisms like *Aspergillus* sp. The older the tea gets, the more smooth taste it develops and the more prominent the color and aroma becomes [30]. However, the process of wet piling is used to convert APT in YPT, which is a process of bacterial and fungal fermentation. Hereby, YPT is piled, dumped, and turned under warm and humid environmental conditions until composting. The two methods of enzymatic treatment change the chemical composition of APT and YPT, significantly.

### 2.2. Time-Temperature Relation with Bioactive Compounds Extraction

The influence of water quality, water temperature, and brewing time on the extraction of bioactive compounds is essential to understand the time-temperature related extraction yield and to establish optimal conditions for the health benefit of the final tea infusion. In general, increase in the infusion time results in an accelerated extraction of bioactive compounds. The present approach is based on the experimental design established by Riehle et al. [31].

In the present study, about 0.4 g of a randomly numbered pu’er tea sample was infused in 40 mL brewing water for 5 min. With regard to the extraction kinetics, samples were collected at 60, 70, 80, 90, and 98 °C. The effect of temperature during a 5 min brewing on TPC and individual bioactive compounds is depicted in Table 5. Redox potential of bioactive compounds was measured using Folin-Ciocalteu assay. 

The present data showed an increasing extraction of bioactive compounds within the indicated temperature range. Herein, compounds were significantly influenced by temperature. As obvious for shu 1, contents of EGC increased during infusion temperature at 80 °C, whereas GCG and CG increased at higher temperatures of 90 °C. Due to a high content of bioactive compounds in sheng 1, all of the known substances are extracted also by lower temperatures. Of note is that tea samples shu 1 and sheng 1 infused at 98 °C produced the most similar content of phenolic compounds than samples infused at 90 °C.

Observations described by Duh et al. [16] could not detect EGCG, ECG or EGC in water extracts of pu’er tea. Hereby, 50 g of tea was extracted with 500 mL boiling water for 5 min. However, an EC content in aqueous extract of pu’er tea was detected with 8 mg/mL. Jeszka-Skowron et al. [32] optimized the extraction process of tea and stated a decreasing process of chemical compounds during fermentation, resulting in a low amount of rutin with 6 µg/mL and a high amount of gallic acid with 94 µg/mL in infusion. For comparison, content of quercetin was found in pu’er tea with 0.4 µg/mL, 5-CQA with 0.5 µg/mL.

In a very recent study, Pérez-Burillo et al. [33] analyzed WT with regard to its sensory properties and its health benefit. Thereby, they analyzed eighty commercial teas for their bioactive compounds (caffeine and individual catechins), different time-temperature ratios, and sensory analysis. In conclusion, brewing at 98 °C for 7 min was the best condition to obtain a high content of antioxidant polyphenols and pleasant sensory properties.

Currently, there is a great demand on the consumption of pu’er tea is because of its health-promoting effect. The difference in preparation and consumption also correlates with drinker’s well-being. Herein, study done by Xie et al. [34] showed that pu’er tea ingestion for two weeks can re-establish gut microbial population. These gut microbes exert a high impact on the development and structure of intestinal epithelium and therefore influences the human metabolism and affect health.

### 2.3. Outcome of Multivariate Data Analysis

**Figure 1:**
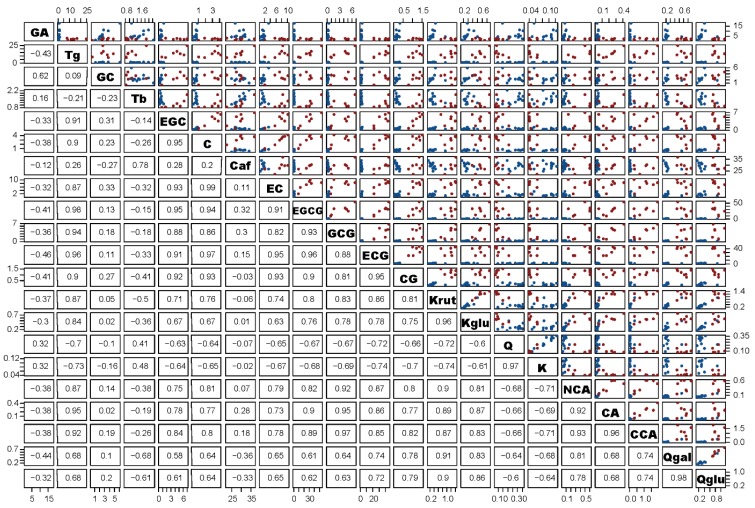
Scatterplot analysis of all analyzed biochemical compounds. Blue spots: phenolic content analyzed in *young ripened* pu’er tea (YPT), red spots: phenolic content analyzed in *aged raw* pu’er tea (APT).

Some of the measured biochemical compounds showed high correlations (Figure 1).

**Figure 2:**
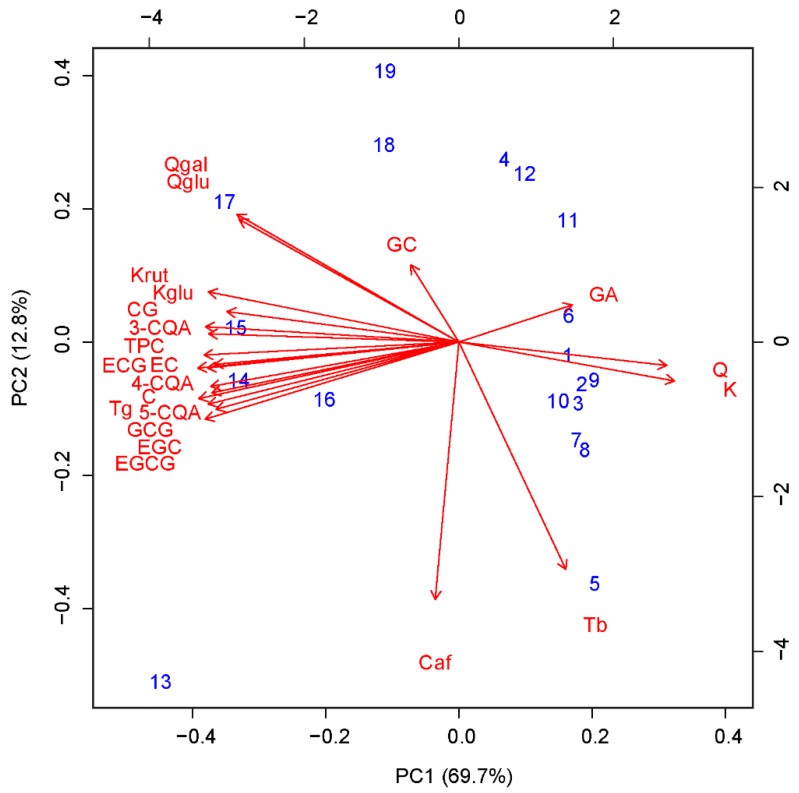
Biplot of the first two principal components of PCA. Number 1–12 refer to scores of YPT samples and 13–19 to scores of APT samples. Red arrows illustrate direction of each analyzed compound on the first two principal components.

The visualization of the principal component analysis (PCA) illustrates commonalities and clustering among the analyzed compounds. Figure 2 shows the biplot graph based on PCA for all analyzed biochemical compounds. The first component explains 69.7% of total variance, the second 12.8%. The examined 19 tea samples can be clearly clustered into two groups: Number 1–12 refer to YPT samples and 13–19 to APT samples. Differentiation between YPT and APT is primarily due to the first principal component. YPT samples are mainly characterized by high GA, Q and K values, and low Q-gal, Q-glu, K-rut, K-glu, CG, 3-CQA, TPC, EC, ECG, CCA, C, Tg, CA, GCG, EGC, and EGCG values. For APT sample, characteristics are the other way round. Ca, Tb and GC loaded more strongly on the second component, which did not result in classification of the two tea types. PCA shows that tea samples from different production years (Table 1), such as shu 1980 and shu 1994 (No. 11 and 12) and sheng 1980 and sheng 1992 (No. 18 and 19) share common characteristics based on chemometric analysis.

In a metabolomics approach, Xie et al. [34] evaluated the quality of GT, BT, and pu’er tea at hand of their chemical constituents. Characteristic polyphenols were theanine in GT, theaflavic acid, and thearubigin in black tea, and theabrownin and gallic acid in pu’er tea.

PCA also helped to distinguish between the seven main consumed tea sorts as for example GT, YT, WT, OT, BT, APT, and YPT. A study done by Yi et al. [9] could identify in particular four main groups of tea. With the first group consisting of BT and YPT, having a common clustering by chemometrics analysis. Wet piling can help to oxidize bioactive compounds for YPT in the same way as bruising for BT. The second group was constituted by the two ‘non-oxidized’ teas, YT and GT. The third group included OT and WT, the two partially oxidized teas. The fourth group was just composed of APT, which shows a complete distinctive composition of bioactive compounds.

A recent study done by Shevchuk et al. [35] on differentiation of black tea infusions describes marker compounds responsible for origin, plant variety, and processing method. Catechins, derivatives of quercetin, apigenin, quinic acid, and kaempferol were there the most variable compounds. In contrary to the present study which shows less variations for the group of APT or the group of YPT, respectively.

**Figure 3:**
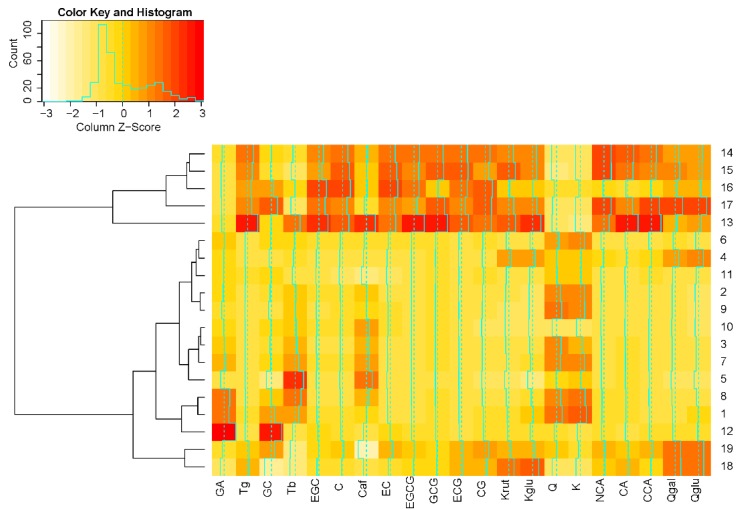
Heat map of hierarchical clustering dendrogram of twenty-one biochemical compounds profiles from pu’er samples obtained by LC/MS. The analyzed samples are characterized both by their biochemical compounds and pu’er tea processing.

The result of the hierarchical cluster analysis (HCA) of the present study is illustrated in Figure 3, whereby the outcome is a dendrogram and a color-coded heatmap with samples grouped in branches according to their similarities.

HCA shows primarily a grouping into two clusters according to processing and year of harvest: APT (No. 13–17) and YPT (No. 1–12), whereby the second cluster also contains sheng 1980 and sheng 1992 (No. 18 and 19). This latter alliance results from a similar polyphenol profile and a lower content of chemical compounds than for APT.

## 3. Materials and Methods

### 3.1. Materials and Reagents

Standards substances of the phenolic compounds were obtained as follows: (+)-catechin, C; (-)-epicatechin, EC; (−)-epicatechin-3-*O*-gallate, ECG; (-)-epigallocatechin, EGC; (-)-epigallocatechin-3-*O*-gallate, EGCG; (+)-gallocatechin, GC; (-)-gallocatechin-3-*O*-gallate, GCG; gallic acid, GA; kaempferol, K; kaempferol-3-*O*-glucoside, K-glu; kaempferol-3-*O*-rutinoside, K-rut; quercetin, Q; quercetin-3-*O*-galactoside, Q-gal; quercetin-3-*O*-rutinoside, Q-rut; 3-*O*-caffeoylquinic acid, neochlorogenic acid, 3-CQA; 5-*O*-caffeoylquinic acid, chlorogenic acid, 5-CQA; and 4-*O*-caffeoylquinic acid, cryptochlorogenic acid, 4-CQA were from PhytoLab GmbH & Co. KG (Vestenbergsgreuth, Germany). Methylxanthines standards like caffeine, Caf; theobromine, Tb; and 3-*O*-galloylquinic acid, 3-GQA, resp. theogallin, Tg, were purchased from Sigma Aldrich Chemie GmbH (Buchs, Switzerland). Acetonitrile, water, and formic acid (LC-MS grade) were purchased from Sigma, as well as 2 M Folin-Ciocalteu reagent and anhydrous sodium carbonate which were used to measure the total phenolic content (TPC). Further reference substances were used for identification but were not detected in pu’er tea: myricetin, theaflavin (TF), theaflavin-3,3-digallat (all from PhytoLab).

The purity of the standards was 98% or higher, and all were prepared as stock solutions at 1 mg/mL in methanol for flavan-3-ols and phenolic acids and DMSO for the flavonols quercetin, kaempferol, etc. The stock solution for theobromine was prepared at 0.2 mg/mL. The stock solutions were stored at −20 °C and used to prepare working mix solutions by diluting the appropriate volume of the stocks in methanol, in order to obtain the desired concentrations for spiking in the range of 0.01 mg/L to 0.4 mg/L.

### 3.2. Plant Material

Five aged raw pu’er teas produced in 2005 and ten young ripened pu’er teas produced in 2016/2017 were obtained from different producing companies in the province Yunnan, People’s Republic of China. In addition, aged raw pu’er tea produced in 1980 and 1992 and young ripened pu’er tea produced in 1980 and 1994 were analyzed in this study. Further details on these pu’er teas are described in Table 1. All of the samples were of proven authenticity and kindly provided by Nannuoshan Tea House (Berlin, Germany). Samples were kept in cloth bags at room temperature in a dry and dark place. In recent years, the pu’er teas are constituted as a single origin, harvested by one single farmer, whereby in the past century blending leaves from different gardens was common.

### 3.3. Sample Preparation

#### 3.3.1. Plant Material

To minimize different extraction kinetics due to different leaf grades, tea leaves were crushed in a knife mill (A 11 basic Analytical Mill, IKA^®^-Werke GmbH & Co. KG, Staufen, Germany). Extraction was done according to Zuo, et al. [36] with some modifications. In the present study, 1 g of the ground pu’er tea leaves were extracted three times with 4 mL 50% aqueous ethanol at 60 °C for ten minutes. In total, 12 mL of the combined polyphenol-enriched supernatant (‘tea leaves extract’) was used for further analysis of single compounds of interest using LC-DAD/ESI-MS and determination of the TPC.

#### 3.3.2. Preparation of the Infusions

Tea consumption differs from country to country. Many herbal teas are made by infusion where tea leaves are stripped in hot water. For the present study, the preparation of tea infusions was done using water with a low degree of total hardness. When tap water would be used for brewing, a lower content on bioactive compounds would be the result. A possible explanation is the so called ‘tea cream’ formation [31], which corresponds with the mineral content in water and forms because of high phenolic content, caffeine and theaflavins, thearubigins, and mineral content of the water. However, optimized brewing conditions of pu’er tea infusions are very important. Here, pu’er tea infusions were prepared according to Riehle et al. [31]. In the present study, 0.4 g whole tea leaves material were weighed into a 50 mL centrifuge vessel and brewed that vessel in 40 mL of distilled boiling water. Infusions were made using in each experimental approach a different time and temperature ratio. Distilled water was heated at 60, 70, 80, 90, and 98 °C and the tea sample was left for 3, 5, 7, 10, and 15 min to obtain the corresponding infusion [33,37]. Each sample was prepared in triplicate and each one was analyzed three times.

### 3.4. Quantification of Chemical Components Using LC-DAD/ESI-MS

Identification of individual compounds of ‘tea leaves extracts’ and tea infusions was confirmed by RP-LC-DAD/ESI-MS. This was performed on an Agilent 1200 series liquid chromatography and quadrupole mass spectrometer with electrospray ionization interface (LC-MS 6120, G6100 series, Agilent Technologies AG, Waldbronn, Germany). The ‘tea leaves’ extracts’ were analyzed using a gradient mixture of water–formic acid (99.9:0.1, *v:v*) (solvent A) and acetonitrile–water–formic acid (94.9:5:0.1, *v:v:v*) (solvent B). A 3.0 × 150 mm Eclipse XDB-C18 (3.5 µm) column (Agilent Technologies AG) was used. The separation was affected using a linear gradient at 38 °C with a flow of 0.25 mL/min as follows: 3% B at 0–5 min, 3–6% B at 5–8 min, 6–11% B at 8–20 min, 11–12% B at 20–25 min, 12–17% B at 25–32 min, 17–20% B at 32–38 min, 20–28% B at 38–44 min, 28–31% B at 44–47 min, 31–38% B at 47–51 min, 38–45% at 51–54 min, 45–50% at 54–58 min, 50–90% at 58–61 min, 90% B at 61–63 min and 90–1% B at 63–64 min.

The samples were analyzed using a full scan from 100–2000 *m*/*z* in positive ionization mode. The comparison of retention times and characteristic fragmentation patterns was done using the aforementioned standard substances. The method was performed using standard substances and afterwards applied to ‘tea leaves extract’ and tea infusion, respectively.

### 3.5. Characterization of Chemical Components Using Folin-Ciocalteu Assay

The advantage of the photometrical Folin-Ciocalteu assay is given by a quick analysis in a crude matrix. The TPC of the tea leaf samples was determined using the Folin-Ciocalteu assay according to Singleton [38]. To obtain a working solution, 2 M Folin-Ciocalteu reagent was diluted in a ratio 1:3 with water. Due to the high TPC content, ‘tea leaves extract’ was diluted four hundred-time, so that finally 200 µL of a the diluted tea extract was mixed with the same volume of Folin-Ciocalteu reagent and left for 3 min. Afterwards, 400 µL of a 20% anhydrous sodium carbonate solution and 400 µL water were added, mixed well and left for 120 min. The absorbance of the reaction mixture was measured at the absorbance maximum (750 nm) using a spectrophotometer (Genesys™ 10S, Thermo Fisher Scientific, Reinach, Switzerland). All samples were carried out in triplicate for each sample (mean ± SD). Gallic acid was used as a calibration standard and final results were expressed as milligrams of gallic acid equivalent per gram (mg GAE/g). The calibration curve of gallic acid was in the calibration range of 10 mg/L to 50 mg/L with a linear regression line of y = 19.41x − 0.0467 with R^2^ = 0.9996.

### 3.6. Statistical Analysis

Data analysis was done using the statistics software R (Version 3.4.3, R Core Team, 2017, R Foundation for statistical computing, Vienna, Austria) [39]. PCA and HCA were applied to detect chemical compounds that best discriminate samples of the two types of tea. Twenty-one chemical compounds were assessed which were obtained from the chromatographic datasets.

PCA is a multivariate technique used to reduce multidimensional data by incorporating the maximum variance present in the data. It can be applied to determine underlying factors that differentiate between groups of data entries. PCA requires a large number of sample sets, whereby within the present study 19 samples were adequate to determine the characteristic differences. For PCA all variables were scaled.

The HCA attempts to group subjects with similar features into clusters and to establish a hierarchy of them. HCA uses scaled variables. The corresponding polyphenol profile dendrogram is sorted row-wise by its unique assignment. The presentation of a hierarchical clustering dendrogram together with a heatmap provides information on characteristic variables for each cluster.

## 4. Conclusions

Pu’er tea is a widely appreciated beverage, which is not only consumed because of its smooth and earthy taste, but also for medicinal purpose. Overall, the results from this study could serve as a reference to traders and consumers when purchasing teas for consumption. The present study shows a fingerprint identification method for biochemical compounds of pu’er tea based on the HPLC profile.

Although APT shows a higher content on most of the analyzed compounds (especially of EGCG > ECG > GCG > 4-CQA, Tg > EGC > CG > 5-CQA > 3-CQA > EC > C > K-rut > Q-glu > Q-gal > K-glu > GC), YPT can still have a profound impact on digestive and absolve capabilities of the intestine. Due to the fermentation-induced structural cleavage, flavonol compounds can released into free aglycons and therefore improving their health benefits. This is the first report describing a difference of flavonols and their corresponding glycosides found in APT and YPT. During the fermentation process, new bioactive compounds are formed due to microbial enzymes which hydrolyse flavonol glycosides and release the corresponding aglycons. Fermentation as an ancient technology assists in improving the bio-accessibility of bioactive compounds and improves their health-linked functionality.

A statistical combination of PCA and HCA was used as appropriate methods to distinguish between pu’er tea varieties and to determine characteristic compounds. Also older pu’er teas could be divided into two clusters. Especially gallic acid, quercetin, and kaempferol were found to be good chemical markers to differentiate between APT and YPT.

The outcome of the present study could be applied for authentication of pu’er teas either to perform quality supervision or to identify food fraud. Future research can focus on developing a fast track analysis for a reliable correlation between specific bioactive compounds and the quality of various pu’er teas. In addition, this research approach may be used for similar natural products, where multivariate factors represent the quality of the product.

Generally, methods for authentication and characterization of different tea varieties have received large attention. Alternative methods with the capability to track the profile of bioactive compounds or to distinguish between different teas varieties are more and more important [40].

## Figures and Tables

**Table 1:** Variety, year of production, region of origin, and company name of the pu’er samples.

No.	Variety	Origin	Name	Year of Production	Producing Company	Storage
1	Shu 1	Menghai (Bulang)	Gaoshan bing	2016	Lang he	n/a
2	Shu 2	Lincang (Fengqing)	Tianlai bing	2016	Long run	n/a
3	Shu 3	Menghai	Dayi 8592	2016	Dayi	n/a
4	Shu 4	Dali	Xiaguan zhuan	2016	Xiaguan	n/a
5	Shu 5	Dali	Jinque bing	2016	Xiaguan	n/a
6	Shu 6	Lincang (Fengqing)	Ruyi bing	2017	Zhong cha	n/a
7	Shu 7	Menghai	Dayi 7572	2017	Dayi	n/a
8	Shu 8	Menghai	Laotongzhi bing	2017	Lao tong zhi	n/a
9	Shu 9	Lincang (Fengqing)	Runxinhao bing	2017	Long run	n/a
10	Shu 10	Pu’er	Lancang bing	2017	Lancang gu cha	n/a
11	Shu 1980	Xishuangbanna	n/a	1980	n/a	n/a
12	Shu 1994	Xishuangbanna	n/a	1994	n/a	n/a
13	Sheng 1	Dali	Canger tuo	2005	Xiaguan	Kunming
14	Sheng 2	Menghai	Changtai bing	2005	Chang tai	Zhejiang
15	Sheng 3	Lincang	Qingzhuan	2005	Bai cha tang	Shanghai
16	Sheng 4	Menghai	Dadugang bing	2005	Da du gang	Zhejiang
17	Sheng 5	Menghai	Ziya bing	2005	Da du gang	Zhejiang
18	Sheng 1980	Xishuangbanna	n/a	1980	n/a	n/a
19	Sheng 1992	Xishuangbanna	n/a	1992	n/a	n/a

n/a not available.

**Table 2:** Mass spectrometric identification results. MS-data and retention times.

No.	Identification	Abbr.	RT [min]	UV [nm]	LOD [mg/L]	LOQ [mg/L]	ATP	YPT	MW [g/mol]	[M + H]^+^ [*m*/*z*]	Major Fragments [M + H]^+^ [*m*/*z*]		
1	Gallic acid	GA	9.4	280	1.6	4.9	x	X	170.12	171.1	153.1	394.0	-
2	Theogallin	Tg	11.4	280	18.2	55.2	X	x	344.27	345.1	153.0	367.0	545.8
3	(−)-Gallocatechin	GC	15.0	280	147	446	X	x	306.27	307.0	139.0	288.7	419.2
4	Theobromine	Tb	15.2	280	4.0	12.3	X	x	180.16	181.1	-	-	-
5	(−)-Epigallocatechin	EGC	22.0	280	49.9	151.3	X	x	306.27	307.1	139.0	629.8	-
6	(+)-Catechin	C	23.8	280	20.6	62.3	X	x	290.27	291.1	123.0	-	-
7	Caffeine	Caf	25.4	280	19.3	58.5	X	x	194.19	195.1	-	-	-
8	(−)-Epicatechin	EC	31.1	280	11.3	34.2	X	x	290.27	291.0	123.1	139.2	165.3
9	(−)-Epigallocatechin gallate	EGCG	31.7	280	6.5	19.6	X	x	458.37	459.1	139.1	289.1	547.1
10	(−)-Gallocatechin gallate	GCG	34.3	280	5.0	15.3	X	x	458.37	459.0	139.0	289.6	884.1
11	(−)-Epicatechin gallate	ECG	40.3	280	13.6	41.2	X	x	442.37	443.1	123.1	139.0	273.1
12	(−)-Catechin gallate	CG	41.1	280	6.9	20.9	X	x	442.37	443.1	-	-	-
13	Neochlorogenic acid	3-CQA	18.2	320	10.0	30.3	X	x	354.31	355.1	163.0	393.1	-
14	Chlorogenic acid	5-CQA	24.2	320	10.6	32.2	X	x	354.31	355.0	163.0	393.1	-
15	Cryptochlorogenic acid	4-CQA	26.1	320	10.1	30.7	X	x	354.31	355.0	163.1	-	-
16	Quercetin-3-*O*-galactoside	Q-gal	42.1	360	18.0	54.6	X	x	464.37	465.1	303.0	-	-
17	Quercetin-3-*O*-glucoside	Q-glu	43.0	360	23.1	69.9	X	x	464.37	465.0	303.0	-	-
18	Kaempferol-3-*O*-rutinoside	K-rut	44.6	360	19.4	58.8	X	x	594.52	595.1	287.1	449.0	-
19	Kaempferol-3-*O*-glucoside	K-glu	45.6	360	19.3	58.6	X	x	448.38	449.1	287.0	-	-
20	Quercetin	Q	52.8	360	2.1	6.3	x	X	302.24	303.0	-	-	-
21	Kaempferol	K	56.9	360	19.3	58.4	x	X	286.24	287.0	-	-	-
22 *	Theophylline	Tp	19.4	280	-	-	-	-	180.16	181.0	124.2	-	-
23 *	Theaflavin	Tf	51.5	280	-	-	-	-	564.49	565.1	121.0	1128.8	1693.3
24 *	Luteolin-6-*C*-glucoside	L-glu	37.6	360	-	-	-	-	448.38	449.1	-	-	-
25 *	Luteolin	L	52.9	360	-	-	-	-	286.24	287.0	-	-	-
26 *	Quercetin-3-*O*-rutinoside	Q-rut	41.9	360	-	-	-	-	610.52	611.0	302.8	465.0	-
27 *	Quercetin-3-*O*-glucuronid	Q-glc	42.6	360	-	-	-	-	478.36	479.0	303.0	-	-
28 *	Apigenin	A	56.5	320	-	-	-	-	270.24	271.0	-	-	-

* The asterisk marks the traces in the mass spectrometer detector.

**Table 3:** Total polyphenol content (TPC) and biochemical composition of APT and YPT. Data are presented as mean ± SD (n = 3) in [mg/g]. The TPC is expressed as milligram gallic acid equivalents per gram sample [mg GAE/g].

No.	TPC	Tg	Caf	Tb	GA	GC	EGC	C	EC	EGCG	GCG	ECG	CG
Shu 1	114 ± 2	1.44 ± 0.1	29.01 ± 0.66	1.73 ± 0.04	10.27 ± 0.26	3.59 ± 0.18	0.74 ± 0.06	0.78 ± 0.01	1.62 ± 0.02	0.89 ± 0.03	0.30 ± 0.00	2.35 ± 0.03	0.29 ± 0.02
Shu 2	99 ± 1	0.80 ± 0.01	29.58 ± 0.05	1.46 ± 0.01	3.97 ± 0.01	2.22 ± 0.01	0.56 ± 0.02	0.71 ± 0.01	1.57 ± 0.01	0.61 ± 0.02	0.19 ± 0.01	1.53 ± 0.02	0.18 ± 0.00
Shu 3	115 ± 4	0.89 ± 0.02	30.96 ± 0.71	1.53 ± 0.04	5.28 ± 0.16	2.19 ± 0.24	0.42 ± 0.02	0.62 ± 0.02	1.18 ± 0.04	0.54 ± 0.02	0.19 ± 0.01	1.76 ± 0.08	0.17 ± 0.04
Shu 4	85 ± 2	0.72 ± 0.01	26.58 ± 0.25	1.18 ± 0.01	4.48 ± 0.02	1.86 ± 0.02	0.44 ± 0.01	0.50 ± 0.01	1.02 ± 0.13	0.50 ± 0.08	0.17 ± 0.00	1.49 ± 0.02	0.14 ± 0.01
Shu 5	83 ± 1	0.88 ± 0.01	34.04 ± 0.28	2.24 ± 0.02	1.07 ± 0.07	1.01 ± 0.02	0.38 ± 0.01	0.44 ± 0.01	0.89 ± 0.11	0.40 ± 0.05	0.11 ± 0.00	1.64 ± 0.03	0.07 ± 0.01
Shu 6	111 ± 1	0.89 ± 0.03	27.36 ± 0.32	1.26 ± 0.02	5.21 ± 0.09	2.28 ± 0.16	0.87 ± 0.02	0.70 ± 0.01	2.03 ± 0.01	1.16 ± 0.04	0.31 ± 0.01	2.28 ± 0.04	0.24 ± 0.01
Shu 7	120 ± 2	1.00 ± 0.01	31.86 ± 0.13	1.72 ± 0.01	6.72 ± 0.04	2.40 ± 0.03	0.69 ± 0.04	0.71 ± 0.01	1.57 ± 0.02	0.67 ± 0.02	0.24 ± 0.01	1.76 ± 0.02	0.16 ± 0.01
Shu 8	116 ± 1	1.28 ± 0.03	31.12 ± 0.39	1.98 ± 0.03	11.18 ± 0.13	2.64 ± 0.11	0.51 ± 0.02	0.68 ± 0.02	1.26 ± 0.03	0.78 ± 0.02	0.23 ± 0.00	1.82 ± 0.05	0.14 ± 0.01
Shu 9	93 ± 2	0.67 ± 0.01	29.18 ± 0.35	1.38 ± 0.02	2.33 ± 0.04	1.70 ± 0.07	0.41 ± 0.01	0.78 ± 0.02	1.75 ± 0.05	0.54 ± 0.01	0.15 ± 0.00	1.60 ± 0.00	0.16 ± 0.01
Shu 10	109 ± 3	0.78 ± 0.01	31.89 ± 0.31	1.47 ± 0.01	4.53 ± 0.11	2.44 ± 0.24	0.64 ± 0.01	0.71 ± 0.02	1.84 ± 0.07	0.47 ± 0.02	0.17 ± 0.00	1.35 ± 0.05	0.06 ± 0.00
Mean Shu 1-10	105 ± 13	0.94 ± 0.25	30.16 ± 2.26	1.60 ± 0.33	5.50 ± 3.17	2.23 ± 0.67	0.57 ± 0.16	0.66 ± 0.11	1.47 ± 0.37	0.66 ± 0.23	0.21 ± 0.06	1.76 ± 0.33	0.16 ± 0.07
Shu 1980	100 ± 2	1.47 ± 0.02	24.83 ± 0.35	1.06 ± 0.01	4.21 ± 0.11	2.44 ± 0.04	0.26 ± 0.03	0.27 ± 0.07	0.48 ± 0.08	0.83 ± 0.05	0.30 ± 0.04	2.22 ± 0.08	0.31 ± 0.03
Shu 1994	148 ± 2	1.07 ± 0.71	26.74 ± 0.28	1.10 ± 0.01	17.81 ± 0.27	5.94 ± 0.10	1.25 ± 0.17	0.86 ± 0.01	3.40 ± 0.03	1.06 ± 0.03	0.42 ± 0.00	3.01 ± 0.04	0.33 ± 0.03
Sheng 1	221 ± 7	24.92 ± 0.67	36.79 ± 0.95	1.87 ± 0.05	2.24 ± 0.07	2.49 ± 0.18	6.94 ± 0.12	3.73 ± 0.45	8.41 ± 0.31	53.94 ± 1.51	6.76 ± 0.57	45.32 ± 1.29	1.35 ± 0.12
Sheng 2	212 ± 7	16.69 ± 0.13	30.40 ± 0.16	1.13 ± 0.01	2.70 ± 0.04	2.60 ± 0.09	4.48 ± 0.07	3.33 ± 0.03	7.98 ± 0.12	34.30 ± 0.32	4.38 ± 0.05	40.77 ± 0.38	1.20 ± 0.06
Sheng 3	206 ± 6	14.16 ± 0.21	29.57 ± 0.40	0.93 ± 0.01	1.88 ± 0.02	2.24 ± 0.03	3.61 ± 0.08	3.82 ± 0.04	9.09 ± 0.11	30.50 ± 0.40	4.70 ± 0.07	44.03 ± 0.65	1.00 ± 0.06
Sheng 4	207 ± 3	11.66 ± 0.17	29.80 ± 0.16	1.33 ± 0.02	1.85 ± 0.10	3.40 ± 0.13	6.19 ± 0.01	4.16 ± 0.06	10.19 ± 0.09	32.24 ± 0.37	1.68 ± 0.02	39.93 ± 0.18	1.51 ± 0.02
Sheng 5	205 ± 10	13.25 ± 0.40	27.36 ± 0.85	0.92 ± 0.02	2.13 ± 0.06	4.61 ± 0.30	5.28 ± 0.26	2.95 ± 0.17	7.27 ± 0.36	24.59 ± 1.13	5.15 ± 0.22	30.64 ± 1.41	1.39 ± 0.13
Mean Sheng 1-5	210 ± 6	16.13 ± 5.24	30.78 ± 3.55	1.24 ± 0.39	2.16 ± 0.35	3.07 ± 0.97	5.30 ± 1.32	3.60 ± 0.47	8.59 ± 1.11	35.11 ± 11.13	4.54 ± 1.84	40.14 ± 5.76	1.29 ± 0.20
Sheng 1980	155 ± 2	8.67 ± 0.21	26.67 ± 0.22	0.82 ± 0.02	0.89 ± 0.07	0.38 ± 0.01	0.80 ± 0.03	1.04 ± 0.02	2.05 ± 0.05	9.25 ± 0.08	0.88 ± 0.01	21.81 ± 0.14	0.79 ± 0.04
Sheng 1992	154 ± 3	7.96 ± 0.05	22.29 ± 0.22	0.84 ± 0.01	3.01 ± 0.06	3.60 ± 0.05	1.72 ± 0.05	1.89 ± 0.02	5.64 ± 0.10	12.71 ± 0.15	1.04 ± 0.00	21.74 ± 0.49	1.03 ± 0.05

**Table 4:** Biochemical composition of APT and YPT. Data are presented as mean ± SD (n = 3) in µg/g.

No.	3-CQA	5-CQA	4-CQA	Kaempferol	K-rut	K-glu	Quercetin	Q-gal	Q-glu
Shu 1	93 ± 2	47 ± 1	32 ± 1	122 ± 3	421 ± 13	422 ± 10	357 ± 12	229 ± 5	440 ± 19
Shu 2	57 ± 1	30 ± 1	35 ± 1	107 ± 1	262 ± 4	223 ± 1	309 ± 6	204 ± 2	208 ± 2
Shu 3	65 ± 2	34 ± 1	23 ± 1	92 ± 1	268 ± 10	247 ± 6	329 ± 7	206 ± 8	302 ± 8
Shu 4	169 ± 19	60 ± 3	345 ± 16	85 ± 1	930 ± 10	497 ± 26	210 ± 2	556 ± 28	871 ± 54
Shu 5	58 ± 1	32 ± 1	30 ± 1	82 ± 1	124 ± 3	167 ± 3	192 ± 4	141 ± 6	138 ± 3
Shu 6	48 ± 1	24 ± 1	20 ± 1	107 ± 1	334 ± 6	271 ± 5	278 ± 2	248 ± 3	259 ± 5
Shu 7	69 ± 1	37 ± 1	25 ± 1	107 ± 2	350 ± 7	290 ± 1	311 ± 14	200 ± 3	287 ± 3
Shu 8	100 ± 1	47 ± 1	28 ± 3	113 ± 1	329 ± 8	314 ± 5	320 ± 2	185 ± 2	317 ± 4
Shu 9	46 ± 1	25 ± 1	36 ± 1	108 ± 1	205 ± 1	227 ± 1	351 ± 6	205 ± 3	224 ± 3
Shu 10	28 ± 2	18 ± 1	20 ± 1	49 ± 2	196 ± 8	213 ± 9	96 ± 3	183 ± 8	199 ± 10
Mean Shu 1-10	73 ± 40	35 ± 13	59 ± 101	97 ± 21	342 ± 224	287 ± 102	275 ± 84	236 ± 116	324 ± 209
Shu 1980	34 ± 2	20 ± 1	48 ± 4	83 ± 2	348 ± 19	255 ± 13	223 ± 7	220 ± 19	291 ± 15
Shu 1994	74 ± 1	30 ± 1	93 ± 3	55 ± 1	462 ± 3	282 ± 7	145 ± 6	244 ± 2	404 ± 1
Sheng 1	497 ± 8	412 ± 10	1902 ± 27	39 ± 1	1428 ± 64	753 ± 25	83 ± 3	464 ± 16	718 ± 24
Sheng 2	605 ± 2	281 ± 1	1178 ± 5	41 ± 1	1190 ± 19	543 ± 5	81 ± 1	545 ± 6	780 ± 26
Sheng 3	609 ± 11	258 ± 4	1117 ± 14	42 ± 1	1394 ± 6	556 ± 6	72 ± 1	616 ± 9	775 ± 8
Sheng 4	147 ± 18	53 ± 2	309 ± 19	65 ± 4	721 ± 37	385 ± 27	148 ± 14	447 ± 27	696 ± 38
Sheng 5	598 ± 21	239 ± 25	1626 ± 45	41 ± 1	1204 ± 56	568 ± 28	102 ± 2	767 ± 30	1132 ± 50
Mean Sheng 1-5	491 ± 198	248 ± 129	1226 ± 606	45 ± 11	1187 ± 282	561 ± 131	97 ± 30	568 ± 130	820 ± 178
Sheng 1980	250 ± 9	163 ± 5	334 ± 11	45 ± 1	1235 ± 7	651 ± 7	94 ± 2	666 ± 5	913 ± 26
Sheng 1992	285 ± 13	119 ± 3	694 ± 15	64 ± 1	903 ± 11	456 ± 7	210 ± 1	654 ± 12	913 ± 17

**Table 5:** Total polyphenol content (TPC) and biochemical composition of APT and YPT depending on the infusion temperature. Data are presented as mean ± SD (n = 3) in [mg/g].

Bioactive compounds	Infusion Temperature [°C]									
	60		70		80		90		98	
	Sheng 1	Shu 1	Sheng 1	Shu 1	Sheng 1	Shu 1	Sheng 1	Shu 1	Sheng 1	Shu 1
TPC [mg GAE/g]	37 ± 1	20 ± 3	40 ± 3	26 ± 2	44 ± 3	32 ± 4	68 ± 6	34 ± 4	71 ± 6	36 ± 2
Gallic acid	1.2 ± 0.1	n.d.	1.7 ± 0.3	0.1 ± 0.0	1.7 ± 0.6	0.1 ± 0.0	2.1 ± 0.1	0.1 ± 0.0	2.1 ± 0.2	0.1 ± 0.1
Theogallin	10.1 ± 1.9	9.8 ± 1.8	12.4 ± 0.6	10.8 ± 0.4	13.6 ± 6.9	12.0 ± 1.5	19.4 ± 1.1	12.7 ± 2.0	19.3 ± 1.3	14.1 ± 4.2
GC	1.1 ± 0.3	2.8 ± 0.3	1.2 ± 0.2	2.9 ± 0.3	3.8 ± 0.1	5.3 ± 0.1	3.9 ± 0.0	5.4 ± 0.4	3.6 ± 0.1	4.7 ± 0.1
Theobromine	0.5 ± 0.1	0.5 ± 0.1	0.6 ± 0.0	0.8 ± 0.1	0.7 ± 0.1	0.7 ± 0.2	1.1 ± 1.0	0.8 ± 0.1	0.9 ± 0.2	0.83 ± 0.0
EGC	1.6 ± 0.3	n.d.	2.4 ± 0.1	n.d.	2.5 ± 0.2	0.8 ± 0.3	3.5 ± 0.7	0.5 ± 0.0	3.4 ± 0.1	0.6 ± 0.0
(+)-Catechin	1.8 ± 0.3	n.d.	1.8 ± 0.4	0.3 ± 0.0	1.8 ± 0.1	0.3 ± 0.1	2.7 ± 0.6	0.4 ± 0.0	2.7 ± 0.1	0.4 ± 0.0
Caffeine	8.6 ± 1.3	9.5 ± 1.5	13.5 ± 2.9	14.2 ± 1.1	15.6 ± 1.3	19.2 ± 2.6	23.9 ± 1.1	21.5 ± 1.5	24.8 ± 2.5	22.3 ± 0.5
(−)-Epicatechin	1.9 ± 0.4	0.5 ± 0.2	2.7 ± 0.2	0.7 ± 0.1	2.7 ± 0.2	0.7 ± 0.2	3.5 ± 1.3	0.8 ± 0.1	3.6 ± 0.6	0.9 ± 0.0
EGCG	6.7 ± 1.8	0.2 ± 0.1	10.4 ± 2.5	0.3 ± 0.1	12.0 ± 0.4	0.6 ± 0.0	18.9 ± 1.9	0.6 ± 0.1	19.4 ± 1.1	0.7 ± 0.0
GCG	1.01 ± 0.2	n.d.	1.41 ± 0.1	n.d.	1.9 ± 0.1	n.d.	2.8 ± 0.3	0.3 ± 0.0	2.4 ± 0.2	0.4 ± 0.0
ECG	5.3 ± 1.2	0.3 ± 0.1	7.5 ± 1.3	0.4 ± 0.1	9.5 ± 0.7	0.4 ± 0.1	15.0 ± 1.9	0.7 ± 0.0	13.9 ± 0.8	0.6 ± 0.5
CG	0.3 ± 0.1	n.d.	0.3 ± 0.1	n.d.	0.7 ± 0.1	n.d.	0.9 ± 0.1	0.5 ± 0.0	0.9 ± 0.1	0.6 ± 0.0

n.d. not detected.

## Abbreviations

(+)-catechin (C), (−)-epicatechin (EC), (−)-epigallocatechin (GC); (−)-gallocatechin gallate (GCG); (−)-epigallocatechin gallate (EGCG); (−)-epicatechin gallate (ECG); (−)-gallocatechin (GC); gallic acid (GA); kaempferol (K); kaempferol-3-*O*-glucoside (K-glu); kaempferol-3-*O*-rutinoside (K-rut); quercetin (Q); quercetin-3-*O*-galactoside (Q-gal); quercetin-3-*O*-rutinoside (Q-rut); 3-*O*-caffeoylquinic acid, neochlorogenic acid (3-CQA, NCA); 5-*O*-caffeoylquinic acid, chlorogenic acid (5-CQA, CA); 4-*O*-caffeoylquinic acid, cryptochlorogenic acid (4-CQA, CCA); caffeine (Ca); theobromine (Tb); theogallin, 3-*O*-galloylquinic acid (Tg); total phenolic content (TPC); high performance liquid chromatography (HPLC); principal component analysis (PCA); hierarchical cluster analysis (HCA); total ion chromatogram (TIC); mass spectrometry detection (MSD); 3-(4,5-Dimethylthiazol-2-yl)-2,5-diphenyltetrazoliumbromid (MTT); aged raw pu’er tea (APT); young ripened pu’er tea (YPT); green tea (GT); yellow tea (YT); white tea (WT); oolong tea (OT); black tea (BT)
